# Use of behavioural and physiological responses for scoring sound sensitivity in dogs

**DOI:** 10.1371/journal.pone.0200618

**Published:** 2018-08-01

**Authors:** Carla Caroline Franzini de Souza, Daniel Penteado Martins Dias, Raquel Nascimento de Souza, Magda Alves de Medeiros

**Affiliations:** 1 Department of Physiological Sciences, Institute of Biological Sciences and Healthy, Federal Rural University of Rio de Janeiro, Seropédica, RJ, Brazil; 2 Graduate Program in Veterinary Medicine, Federal Rural University of Rio de Janeiro, Seropédica, RJ, Brazil; 3 Graduate Program in Physiological Sciences, Federal Rural University of Rio de Janeiro, Seropédica, RJ, Brazil; 4 Barão de Mauá University Center, Ribeirão Preto, SP, Brazil; Faculty of Animal Sciences and Food Engineering, University of São Paulo, BRAZIL

## Abstract

Sound sensitive dogs have exaggerated responses to sound stimuli that can negatively impact the welfare of the dog. Behavioural reactions combined with the response to sound involve a marked autonomic imbalance towards sympathetic predominance and release of cortisol. The purpose of the present study was to evaluate, in the laboratory, the cardiac autonomic modulation using heart rate variability (HRV) analysis, serum cortisol levels and behavioural parameters in response to sounds of fireworks in dogs with a history of sensitivity to fireworks. Based on these data, and combining qualitative measures and categorical measures, we propose one short and one full index of sound sensitivity in dogs. Six privately owned dogs with no history and another twelve dogs with a history of sound sensitivity to fireworks were used. The sound stimulus consisted of a standardised recording of fireworks (180-seconds long) with a peak intensity of 103–104 dB. The cardiac intervals were recorded using a frequency meter (Polar® RS800CX model) to evaluate the HRV, and the acquired data were processed using CardioSeries 2.4.1 software. Twenty-one behavioural parameters were analysed quantitatively by time, frequency or categorically by scores and were grouped in behavioural categories of arousal, fear, relaxation and “other”. Sound sensitive dogs had exacerbated autonomic responses to the sound stimulus in the laboratory compared to non-sensitive dogs, with higher LF/HF ratios suggesting autonomic imbalance towards sympathetic predominance, but the cortisol levels were similar between the sensitive and non-sensitive dogs. Sound sensitive dogs showed pronounced responses for the parameters: alert and attention, search sound, startle, trembling, hiding, run away and less intense responses for the parameters rest and wink/sleep. Furthermore, the behavioural categories of arousal, fear, relaxation (lack of) and LF/HF were correlated to the caregiver’s perception of the sound sensitivity of the dogs. Not only the short index for sound sensitivity (behavioural categories arousal, fear and relaxation, and LF/HF ratio) but also the full index for sound sensitivity (all behavioural categories, LF/HF and cortisol levels) was highly correlated to sound fear response at home. These indexes can contribute to the development of strategies to treat sound sensitive dogs.

## Introduction

Loud and sudden sounds can induce a wide range of fearful behaviours in dogs, ranging from evidence of minor anxiety to quite marked behaviours. Although the fear response is a natural and self-protecting behaviour, in sound sensitive fearful dogs these responses are exaggerated and inappropriate and negatively impact the welfare of the dog [1]. Sound sensitive dogs may show a variety of signs such as restlessness, panting, increased startle response, trembling, hiding, arched posture, salivation, destructiveness, defecation, vocalisation, self-mutilation, among others [2]. Sound sensitivity in dogs is a widespread condition [3] and is frequently associated to other behavioural problems and might cause extensive damage to property and could be harmful to the dog itself, to people and other dogs [4, 5].

Besides the behavioural reaction, sound stimuli may cause marked autonomic imbalance towards sympathetic predominance and conspicuous cortisol release [6]. The exaggerate autonomic activation, and cortisol release can ultimately lead to reduced immunity and increased risk for conditions like hypertension, heart disease, fatigue and insomnia [7, 8]. The diagnosis of sound sensitivity in dogs can be quite inaccurate since it relies on owner perceptions regarding the reactions of the dog when facing a noisy situation at home. Therefore, precise measurements of physiological parameters should be considered to develop reliable methods of diagnosis and evaluation of this condition (i.e. high sensitivity to sounds), and the assessment of the efficacy of therapeutic approaches [5].

Previous studies have analysed the behavioural response to fireworks or thunderstorm stimuli in sound sensitivity dogs in a domestic environment and assigned scores to several behaviours such as trembling, vocalisation, salivating, destruction, search for people and running around [9–11]. On the other hand, others have analysed dogs that are non-sensitive to sounds, regarding behavioural responses alone [12, 13] or behavioural and physiological responses combined [14–16]. However, to the best of our knowledge, no study has coupled behavioural, autonomic and endocrine aspects together to propose a single index for sound sensitivity in dogs.

The purpose of the present study was to evaluate, in a laboratory environment, the cardiac autonomic modulation using heart rate variability (HRV) analysis, serum cortisol levels and several behavioural parameters in response to recorded sounds of fireworks in dogs with a history of exaggerated fear of fireworks. Then by combining the qualitative measures of HRV and cortisol data, with the qualitative and categorical measures of behavioural data, we propose two sound sensitivity indexes (one short and one full) for dogs.

## Methods

All procedures were assessed and approved by the ethics committee on animal use of the Institute of Biological Healthy Sciences of the Federal Rural University of Rio de Janeiro (UFRRJ), RJ, Brazil/ CEUA-ICBS, protocol n° 23083.4796/2014-14 and COMEP-UFRRJ n° 23083. 007539/2013-45.

### Animals

Dogs of both sexes, 2 to 6 years old, weighing from 10 to 30 kg and in good health were recruited through an advertisement placed at the Veterinary Hospital for Small Animals and other facilities of the UFRRJ (Seropédica, RJ, Brazil). The ad was also released onto social media linked to the university community. Animals with signs or history of neurological problems and aggression were excluded from the study. The recruitment period lasted from October 2014 until February 2015.

The owners filled out a questionnaire regarding the daily routine of their dogs (S1 and S2 Appendices). and their reactions to sounds (adapted from [4, 11]) (S3 and S4 Appendices). The perception of the caregiver to responses such as panting, trembling, hiding, search for caregiver, restlessness, vocalisation, destructive attitude, excessive salivation, inappropriate elimination and self-trauma were recorded in scores ranging from 0 (absence of the behaviour) to 5 (frequently observed or intense behaviour). Dogs were considered sensitive to sounds when, during fireworks stimulus, scores ≥3 in at least three behavioural parameters were seen or when one behavioural parameter alone reached the maximum score (5). Dogs were also considered sensitive to sounds when their behaviours put their health and physical integrity at risk or when the reaction to sound was very frequent (observed more than 70% of the exposures to sound stimuli). The sum of the scores of each response (panting, trembling, hiding, search for caregiver, restlessness, vocalisation, destruction, salivation, elimination, auto-mutilation) resulted in a score which relies on the perception of the caregiver to the reactions of the dogs to sounds. Considering the importance of dog-human interactions and the previous history of the dog in the behavioural response, the caregivers also answered a questionnaire about their home environment and the interaction of the dogs with humans and other dogs.

### Experimental design

On the day of the sound test, all individual experimental procedures started at 0900h. First, with the dogs (n = 6, non-sensitive dogs; n = 12, sound sensitive dogs) at their respective houses, a non-invasive elastic band containing a contact electrode (Polar RS800cx, Polar®, Kempele, Finland) was trapped to the chest of the dogs in order to have heart rate (HR) sampled on a beat-by-beat basis. Then, the dogs were left undisturbed for 10 minutes for baseline measurements of HR (Basal1) and were subjected to baseline collection of blood samples (SB1). Next, the dogs were taken to the test room by car (a 10 to 15-minute-trip) in their transport boxes.

At the test room, the dogs were left to rest undisturbed for 30 minutes, and an additional baseline measurement of HR was made (Basal2), followed by the second blood sample collection (SB2). Animals were then placed 1 meter away from the sound source and exposed to a 180-second-long recording of fireworks acquired from the website http://www.sound-effect.com. The sound level was adjusted to 103–104 dB and tested with a decibel meter (Sound Level Meters, Model 732A, BK Precision®, Yorba Linda, CA, USA).

**Fig 1:**
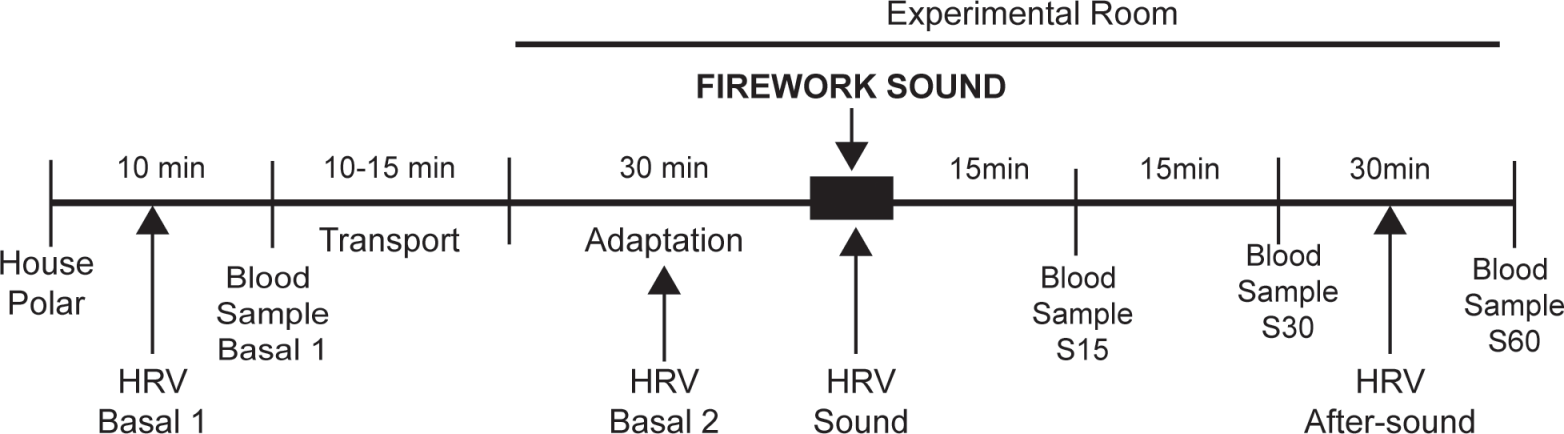
Experimental protocol.

Other blood samples were collected 15 (S15), 30 (S30) and 60 (S60) minutes after the end of the sound stimulus. To assess behavioural responses, dogs were videotaped during the whole sound stimulus period and the 5 minutes following the sound stimulus. Finally, the HR monitors were removed, and the dogs were returned to their houses (Fig 1).

A detailed description of the experimental procedure and laboratory environment was described elsewhere [6].

### Cardiac interval variability analysis

The procedures for the cardiac interval variability (CIV) analysis in response to sound in dogs was previously described [6]. Briefly, HR data were continuously acquired through the cardiac monitor (RS 800cx, Polar, Kempele, Finland) and the data were transmitted from the HR monitor to a custom computer software (Polar Pro Trainer v5, Polar, Kempele, Finland) via an infrared interface. The time series of cardiac interval values from the moments *Basal 1* (300-seconds-long; at the house), *Basal 2* (300-seconds-long; at the test room), *Sound* (180-seconds-long; during sound stimulus) and *After-sound* (300-seconds-long; 30 minutes after the end of sound stimulus) were analysed.

CIV analysis in the time-frequency domain was performed using the freely-available computer software (CardioSeries v2.4.1—http://www.danielpenteado.com). For the time domain analysis, the square root of the mean of the sum of the squares of differences between adjacent cardiac intervals (RMSSD) and the ratio between the standard deviation of cardiac interval values (SDNN) and the RMSSD (SDNN/RMSSD) were calculated following processing of original beat-by-beat series with cardiac interval values [17, 18]. For further frequency domain analysis, the beat-by-beat time series of cardiac interval values were converted to data points every 250 ms using cubic spline interpolation (4 Hz) and divided into half-overlapping sequential sets of 512 data points which were detrended by subtracting the linear trend (obtained by linear regression calculation) from the data points. Next, a well-experienced researcher visually inspected the data points (i.e. cardiac interval values) searching for transients that could affect the power spectral density (PSD) calculation. To confirm that the visual inspection of the cardiac interval time series was adequately performed, a Hanning window was used to attenuate the side effects, and the spectrum of the segments was calculated using a direct fast Fourier Transform (FFT) algorithm, followed by visual inspection for abnormal spectra. Noisy segments were not taken into consideration for analysis. The spectra were integrated into the low-frequency band (LF, 0.04–0.15 Hz) and high-frequency band (HF, 0.15–0.40 Hz). The normalised values were obtained by calculating the percentage of LF and HF power with regard to the total power of the spectrum minus the very low-frequency band (VLF, <0.04Hz) power [19, 20]. The LF/HF ratio was calculated and considered as an index of the sympathovagal balance [21].

### Blood samples, cortisol analysis

Serum cortisol concentrations were determined from blood serum by a double antibody radioimmunoassay method using a commercial kit (RD Coated Tube Cortisol I125 RIA, Costa Mesa, CA, USA), with an assay sensitivity of 0.17 μg/dL and an intraassay coefficient of variation of 6.59%. The blood samples were collected from the cephalic vein using sterilised, intravenous, disposable needles 22G and sample tubes. The tubes were centrifuged for 10 minutes at 3200 rpm to obtain the serum which was stored in plastic containers at -20°C.

### Behavioural analysis

The behavioural responses of the dogs were analysed from videotape recordings (Digital movie camera Sanyo C40, Moriguchi, Osaka, Japan) at the *Baseline* (2 min), *Sound Stimulus* (2.5 min) and *After-Sound* (2 min) moments. Video recordings of each test session were analysed for behavioural signs without sound by a single trained observer (CCFZ) who was blinded to the dog group. Twenty-one behaviours were measured according to the nature of each response, by time (in seconds), frequency (number of occurrences) or by intensity (six points scale: 0 = no signs, 1 = mild and occasional signs; 2 = occasional and moderate/some of the time and mild signs; 3 = most of the times and mild/some of the time and modest signs; 4 = some of the time and severe/ most of the time and moderate signs; 5 = most of the time and intense signs) [15]. Table 1 details the behavioural parameters assessed.

**Table 1:** Description of 21 behavioural parameters examined.

PARAMETERS	Type of analysis	DESCRIPTION
**Arousal**		
Alert and attention	T	Awareness of the environment and the potential presence of danger, orientation towards every sound or event in the environment
Panting	T	An increased frequency of inhalation and exhalation often in combination with opening the mouth
Ambulation	F	Repeated movements (walking, sitting, getting up) without a specific goal and without actually running
Search sound	F	Visual and auditory search for the origin of the sound, with ear and head movements
Startle	I	An exaggerated response to fright, jump in any direction in response to the sound
**Fear**		
Trembling	T	Clear shivering or tremor (quick, short and somewhat rhythmic movements) of one or more body parts
Whine	T	Whines
Tail between legs	T	Shrinking of the back of the body, with tail gathering between the hind limbs
Arched posture	T	Shrinkage of the whole body, accompanied by a low head
Runaway	F	Intentionally trying to get out of the place, orientation towards to the door or other possible exits
Hiding	F	Searches for places to hide, such as behind and under furniture, but stays in the room
Freezing	I	An involuntary reaction of paralysis of the whole body, accompanied by wide open and static eyes
**Relaxation**		
Rest	T	Relaxed body sitting or lying down, with no focus on the environment
Wink/ sleep	F	Deep relaxation to the point of blinking or dozing
Wagtail	F	Fast and wide-open movements, swinging at hip height
**Other**		
Yawn	F	Involuntary movement of inspiring a significant amount of air through the mouth opening
Bark	T	High and directed canine vocal sound
Growl	T	Emission of low, threatening sound between the teeth
Elimination	F	Defecation, urinating, vomiting, diarrhoea
Lip Lick	F	Clearly increased salivation or increased saliva swallowing frequency
Destruction	I	Tries to dig or scratch the floor or bite objects in the room.

The behavioural parameters in response to the fireworks sound stimulus were measured by time (T), frequency = number of occurrence/time (F) or categorised on a scale of four grades analysed: by intensity (I) of behavioural response, adapted from [6, 13].

The behavioural parameters were assigned to four general categories of reaction frequently associated with response to sound: a) behaviours of *arousal*, including Alert and Attention, Panting, Ambulation, Search sound, Startle; b) *fear* behaviours, including Trembling, Whining, Tail between legs; Arched posture; Running around, Hiding and Freezing; c) behaviours related to *relaxation* and "well-being" that is reduced during stress, including Rest, Wag tail, and Blink sleep; d) *other* behaviours that are not actively associated with any of the previous categories: Yawn, Bark, Growl, Elimination, Licking lips, Destruction. The grouping of behavioural parameters into the categories was related to the primary neurobiological or psychobiological meaning of each behavioural parameter.

### Building the full and short indexes for sound sensitivity

To create a sound sensitivity index, the quantitative and qualitative measures were combined [22, 23]. Initially, the quantitative measures of LF/HF ratio (at the moment of the sound) and cortisol levels (15 min after the sound stimulus) and the quantitative measures of each behavioural reaction (at the moment of the sound) was transformed into a six-point scale proportional to the magnitude of the response. The behavioural parameters measured as the duration of the behaviour were converted into a percentage scale of time spent in that behaviour during the period analysed (180 s). As the parameters measured by frequency had a more significant variation, specific scales were attributed to each parameter. The parameters examined by intensity were already in a six-point scale (see Table 2 for details).

**Table 2:** Scoring the sound sensitivity in dogs.

	Score						
	0	1	2	3	4	5	
LF/HF at sound	0	0.1–2.5	2.6–3.0	3.1–3.5	3.6–4	> 4.0	Score of LF/HF
Cortisol increase	> 100	100–175	176–225	226–275	276–325	> 326	Score of cortisol
**Arousal**							
Alert and attention	0	< 20%	21–40%	41–60%	61–80%	> 81%	Average Score of Arousal
Panting	0	< 20%	21–40%	41–60%	61–80%	> 81%	
Ambulation	0	1–2	3–4	5–6	7–8	≤ 9	
Search sound	0	1–2	3–4	5–7	8–10	≤ 11	
Startle	0	1	2	3	4	5	
**Fear**							
Trembling	0	< 20%	21–40%	41–60%	61–80%	> 81%	Average Score of fear
Whine	0	< 20%	21–40%	41–60%	61–80%	> 81%	
Tail between legs	0	< 20%	21–40%	41–60%	61–80%	> 81%	
Arched posture	0	< 20%	21–40%	41–60%	61–80%	> 81%	
Run away	0	1–2	3–4	5–7	8–10	≤ 11	
Hiding	0	1	2–3	4–5	6	≤ 7	
Freezing	0	1	2	3	4	5	
**Relaxation**							
Rest	0	< 20%	21–40%	41–60%	61–80%	> 81%	Average score of relaxation = lack of relaxation^a^
Wink/ sleep	0	1–2	3–4	5–7	8–10	≤ 11	
Wagtail	0	1	2–3	4–5	6	≤ 7	
**Other**							
Yawn	0	1	2	3	4	≤ 5	An average score of other Behaviours
Bark	0	< 20%	21–40%	41–60%	61–80%	> 81%	
Growl	0	< 20%	21–40%	41–60%	61–80%	> 81%	
Elimination							
Lick Lips	0	1–2	3–4	5–7	8–10	≤11	
Destruction	0	1	2	3	4	5	
Total							

Parameters analysed quantitatively were converted to a six-point scale.

^a^ Relaxation was transformed into a lack of relaxation, subtracting five from the average score of the relaxation category.

Next, the correlations between the perception of the caregiver of the sound sensitivity of his/her dog and the scores of the behavioural categories arousal, fear, relaxation and "other" (average of the behavioural scores included in each the category), LF/HF and cortisol were calculated. As all variables are directly proportional to the increase of the response intensity, the category relaxation was transformed into a lack of relaxation, by subtracting five from the average score of the relaxation category. Afterwards, two indexes of sound sensitivity were created: the full index for sound sensitivity, considering the average score of all behavioural categories, LF/HF and cortisol and the short index for sound sensitivity considering only scores strongly correlated with the caregiver’s perception of the dogs’ sound sensitivity. Table 2 shows how the quantitative data were transformed into categorical data to create the indexes of sound sensitivity.

### Statistical analysis

Statistical analyses were performed with SPSS Version 21 (IBM SPSS Inc., Chicago, IL, USA) using a two-way-random-model (confidence-interval 95%). Graphs were built using GraphPad Prism 5.0 (GraphPad Software). Data are expressed as mean ± error.

To compare the response to sound stimulus between non-sensitive and sound sensitive dogs, data of HRV, cortisol and behavioural parameters measured by time and frequency were analysed by two-way ANOVA for the repeated-measures approach followed by Bonferroni’s multiple comparisons (factors time, group (non-sensitive and sound-sensitive), sex, age, weight and neutered) and repeated measures for the factor time: *Basal 1*, *Basal 2*, *Sound* and *After Sound* for HRV parameters; and *Basal 1*, Basal 2, S*15* and S*30* for cortisol, basal, sound and after-sound for behaviours). Due to the significant difference between the groups in the basal levels, for cortisol, HR, RMSSD and SDNN the data baseline values (*Basal 1*) were used as a covariate. Behavioural parameters measured by intensity were analysed by non-parametric testing (Kruskal-Wallis).

The average score of the behavioural categories, LF/HF and cortisol, were correlated with the general score of sound fear at home (owner's perception of the dog's reaction to fireworks at home) by the Spearman test.

## Results

Thirty-six owners answered the advertisement, and 12 companion dogs with an exaggerated fear of sounds and six dogs with no history of sensitivity to sound were included in the present study. The non-sensitive dogs, 3.33±1.03 years old, consisted of three males and two non-neutered females and one neutered female, weighing 20.67±7.42Kg. The sound sensitivity group included five non-neutered and four neutered females and one non-neutered and two neutered males, 3.33±1.37 years old and weighing 17.17±7.45Kg. No differences were found between sound sensitive and non-sensitive dogs regarding sex, age, weight and castration. The perception of the caregiver of the dog for sound sensitivity varied between 0 and 2, with an average value of 0.8±0.9 in the non-sensitive dogs and 15 and 28, with an average value of 21.62±3.98 in the sound sensitive dogs (Supplementary data). All animals selected for the study had reasonably similar management, feeding and family environment. The animals were fed on commercial feed and lived in homes with unrestricted grounds and slept outdoors in their own space with adequate shelter. All dogs included in the current study were left alone at home during specific periods of the day and most had the company of other animals (S5 Appendix).

**Fig 2:**
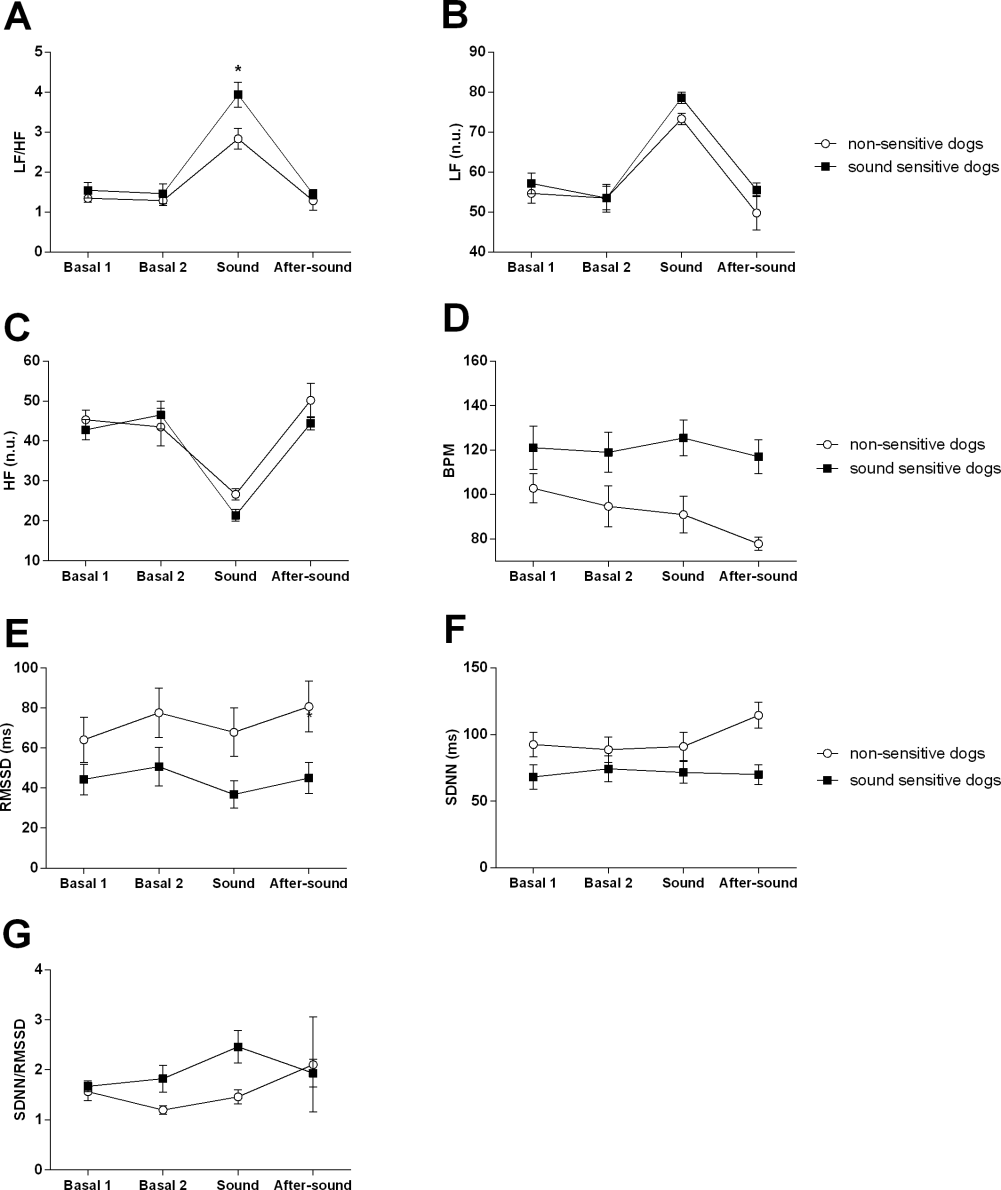
Effect of fireworks sounds on the cardiac interval variability, examined through frequency domain analysis, and heart rate in sound sensitive dogs and non-sensitive dogs. The ratio between the power of the low and high frequency bands of the cardiac interval spectrum (LF/HF, Panel A), power of the LF (Panel B) and HF (HF, Panel C) bands, heart rate (HR, Panel D), the square root of the mean of the sum of the squares of differences between adjacent cardiac intervals (RMSSD, Panel E), standard deviation of cardiac interval values (SDNN, Panel F) and the ratio between the RMSSD and SDNN (SDNN/RMSSD, Panel G). Data obtained at Basal 1 (dogs at the house), Basal 2 (dogs in the test room), Sound (during the acoustic stimulus) and After-Sound (30 min after the end of the sound). * P<0.05 compared to non-sensitive dogs. Data are shown as the mean ± standard error of the mean.

The repeated measures ANOVA detected that the sound of thunder promoted an increase in the power of the LF band of the cardiac interval spectrum and in the LF/HF ratio and a decrease in the power of the HF band of the cardiac interval spectrum, (LF/HF: Wilks' lambda = 0.173, F_[3,14]_ = 22.234, p < 0.0001; LF: Wilks' lambda = 0.891, F_[3,14]_ = 37.965, p < 0.0001, HF: Wilks' lambda = 0.891, F[3,14] = 37.962, p < 0.0001). The sound stimulus did not significantly change the HR, RMSSD, SDNN and SDNN/RMSSD values. Independent of the time point (i.e. Basal 1, Basal 2, Sound, and After-Sound) sound sensitive dogs showed higher HR (Group factor: F (1,15) = 6.594, p = 0.021). The fireworks sensitive dogs showed higher LF/HF during the sound stimulus (Bonferroni test: p = 0.035) (Fig 2).

**Fig 3:**
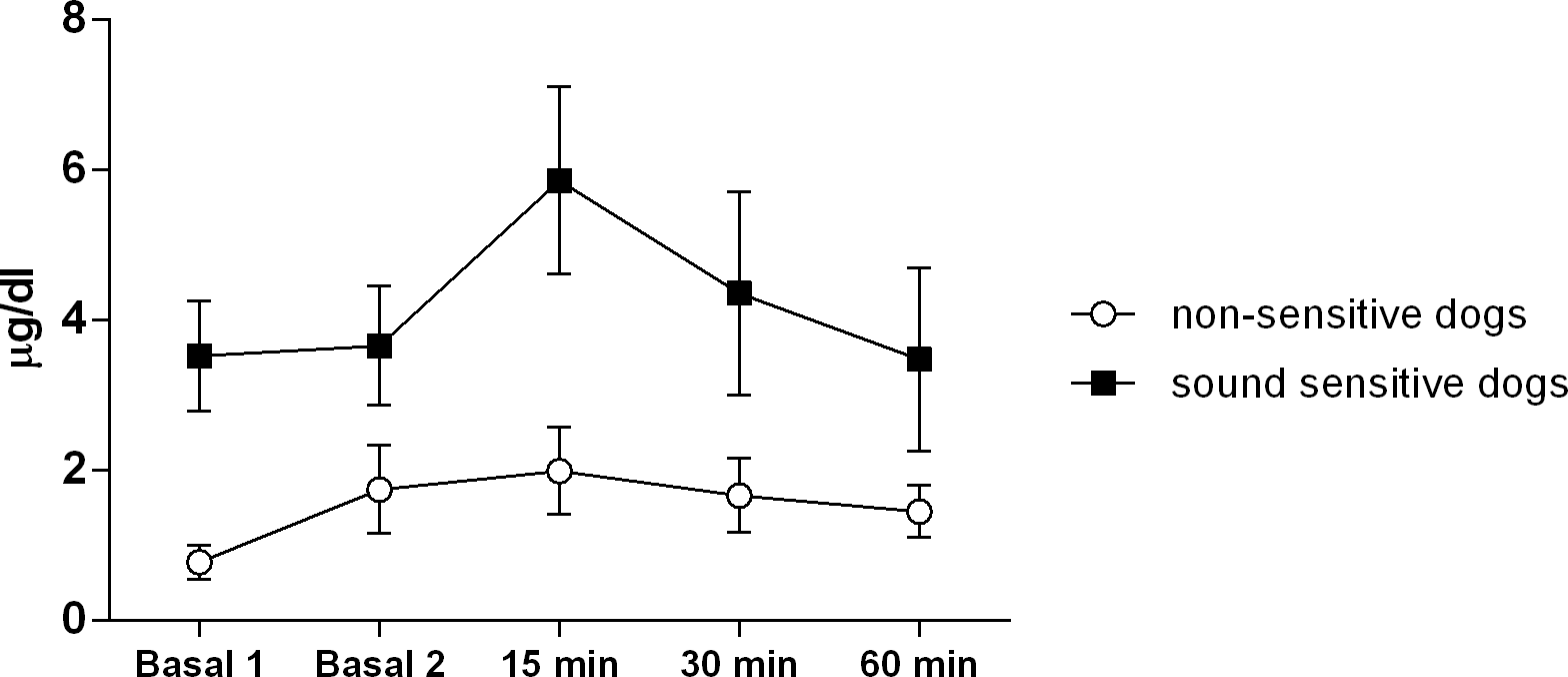
Changes in serum cortisol in response to the fireworks sounds in fireworks sensitive dogs and non-sensitive dogs. Data obtained from Basal 1 (dogs at the house), Basal 2 (dogs in the test room), 15, 30 and 60 minutes after the end of the fireworks sound. Data are shown as media ± error.

Repeated measures ANCOVA also compared the effect ‘*animal group’* on cortisol levels at different time points after the sound stimulus, using Basal 1 as a covariate to account for any potential initial differences. The sound stimulus increased the cortisol levels (Wilks’ lambda = 0.803, F (4,10) = 10.167, p = 0.002), and although the cortisol values showed an apparent difference between fireworks sensitive and non-sensitive dogs, no statistical difference between sensitive and non-sensitive dogs (no effect of group and group x time) was observed (Fig 3).

Table 3 shows the behavioural parameters in response to fireworks of the sound sensitive and non-sensitive dogs. The sound stimulus increased, regardless of the group, in the behaviours: Alert and Attention (Wilks’ lambda = 0.726, F(2,15) = 19.862, p = 0.001); Panting (Wilks’ lambda = 0.349, F(2,14) = 3.751, p = 0.049); Search sound (Wilks’ lambda = 0.549, F(2,14) = 9.145, p = 0.003); Startle (Kruskal Wallis test, p< 0.0001); Trembling (Wilks’ lambda = 0.549, F(2,14) = 6.161, p = 0.011); Hiding (Wilks’ lambda = 0.663, F(2,14) = 3.816, p = 0.046) and run away (Wilks’ lambda = 0.640, F(2,14) = 4.211, p = 0.035). The behavioural analysis shows that the sound sensitive dogs had more intense responses to sound in the parameters Alert and attention (p = 0.001), Search sound (p = 0.036), Trembling (p = 0.009), Hiding (p = 0.040) and run away (p = 0.039) and less intense response in the parameters Rest (p = 0.0001) and Wink/sleep (p = 0.0001). Other behavioural parameters were not statistically different between sound sensitive and non-sensitive dogs.

**Table 3:** Behavioural parameters in response to firework in non-sensitive and sound sensitive dogs.

	Non-sensitive			Sound-sensitive		
PARAMETERS	Basal1	Sound	After-sound	Basal1	Sound	After-sound
**Arousal**						
Alert and attention #	8.5±14.37	22.33±5.99	7.67±6.53	19.91±29.54	101.67±46.52*	11.50±17.66
Panting #	19±26.87	63.4±71.92	22.2±23.58	59.67±64.73	79±61.02	59.33±70.41
Ambulation	1±1.09	1.8±1.72	1.33±2.16	2.5±2.84	4.5±2.93	0.83±1.27
Search sound #	0±0	2.83±1.47	0±0	0±0	8.16±5.52*	0.83±0.28
Startle #	0±0	2.0±0.63	0±0	0±0	2.75±0.62	0±0
**Fear**						
Trembling #	0±0	6.5±10.87	0±0	0±0	63.17±45.81*	5.25±8.13
Whine	0±0	0±0	0±0	0.41±1.44	2.33±4.05	6.58±20.02
Tail between legs	0±0	0±0	0±0	0±0	2.16±5.07	0±0
Arched posture	0.67±1.63	0±0	0±0	8.33±28.86	20.58±42.9	2.33±5.71
Runaway #	0±0	0.17±0.41	0±0	0±0	3.91±4.01*	0.25±0.62
Hiding #	0±0	0.33±0.82	0±0	0±0	3.00±2.82*	0.16±0.57
Freezing	0±0	0±0	0±0	0±0	0.50±0.79	0.33±0.65
**Relaxation**						
Rest	47.33±13.67	53.67±21.75	46.33±13.09	57.67±53.42	9.25±14.08*	76.16±78.19
Wink/ sleep	3.5±2.58	6.17±4.7	3.33±4.17	2.83±2.94	0±0 *	3.7±3.64
Wagtail	1.5±2.3	2±2.75	4.67±9.1	0.25±0.62	0.41±1.44	0.16±0.58
**Other**						
Yawn	0.16±0.41	0.67±1.21	0±0	0.41±0.66	0.58±1.16	0.5±0.79
Bark	0±0	0±0	0±0	0.58±2.02	0.5±1.73	0±0
Growl	0±0	0±0	0±0	0±0	0±0	0±0
Elimination	0±0	0±0	0±0	0±0	0±0	0±0
Lick Lips	2.17±1.47	2.67±1.75	0.67±1.032	2.5±3.14	7.67±7.1	2.16±1.85
Destruction	0±0	0±0	0±0	0±0	0±0	0±0

* indicates a difference between sound sensitive and non-sensitive dogs at the moment of sound

# indicates the parameters with significant effect of sound (effect time independently of the group).

**Fig 4:**
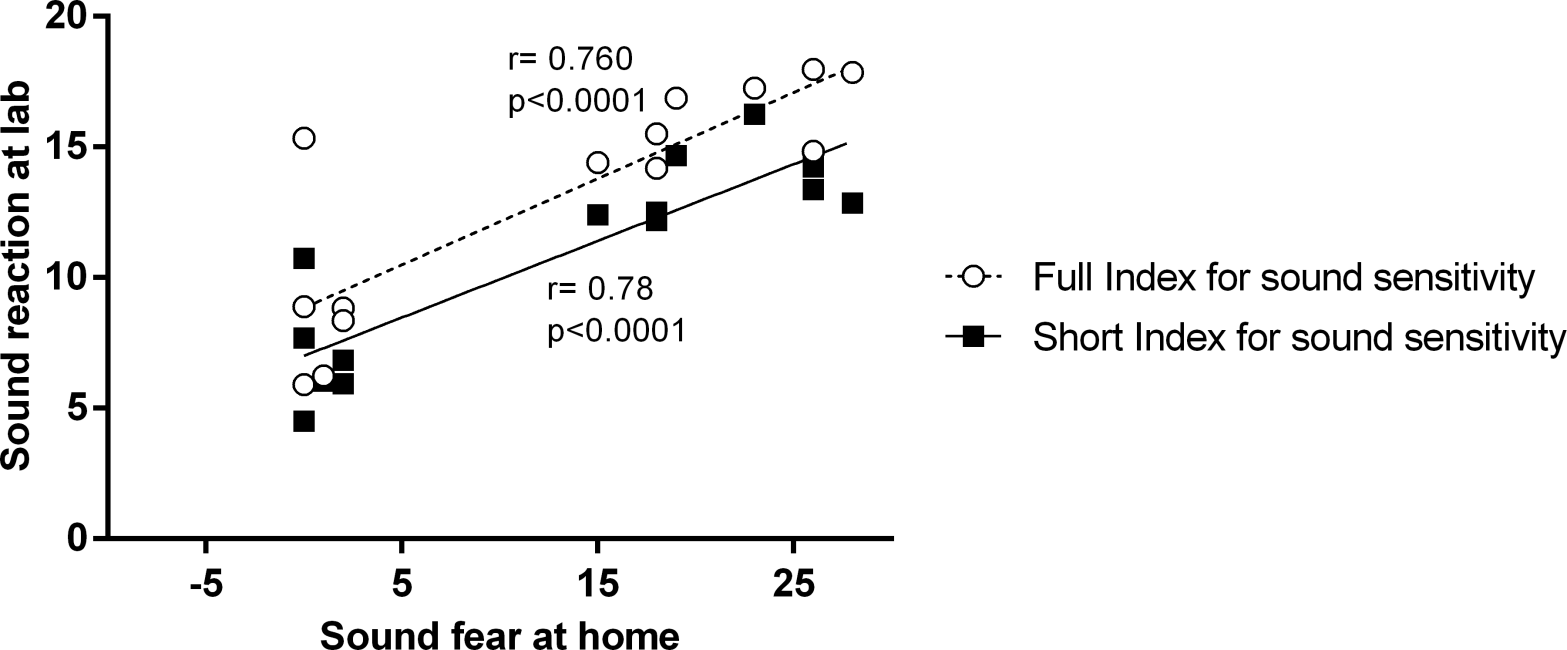
Indexes for sound sensitivity in dogs. Correlation between the perception of the caregiver of the dog’s sound sensitivity and the full index for sound sensitivity (when considering all behavioural categories, LF/HF, cortisol scores) and short index for sound sensitivity (considering behavioural categories of arousal, fear, lack of relaxation and LF/HF score). Data of all animals see S1 Dataset.

The Spearman’s test shows a significant correlation between the perception of the caregiver of the dog’s sound sensitivity and the behavioural categories arousal (r^2^ = 0.57, p = 0.002), fear (r^2^ = 0.771, p = 0.002), lack of relaxation (r^2^ = 0.716, p = 0.002) and LF/HF (r^2^ = 0.523, p = 0.03). Both full index for sound sensitivity (when considering all behavioural categories, LF/HF and cortisol scores) and short index for sound sensitivity (considering only correlated categories: arousal, fear, lack of relaxation and LF/HF score) were correlated to sound fear responses at home (r^2^ = 0.750, p = 0.001 and r^2^ = 0.78, p<0.0001; respectively) (Fig 4).

## Discussion

Our findings indicate that sound sensitive dogs had exacerbated autonomic and behavioural responses to the sound stimulus in the laboratory compared to non-sensitive dogs. Sound sensitive dogs had a more pronounced increase of LF/HF ratios that suggests autonomic imbalance towards sympathetic predominance and more intense response in the behavioural parameters related to arousal and fear and less intense response in behaviours related to relaxation. Although the sound-induced cortisol levels increased in both groups, there were no significant differences between sensitive and non-sensitive dogs.

The present study analysed changes in behavioural responses, serum cortisol levels and HRV parameters in dogs subjected to sound stimulus in a laboratory setting. Although there are differences between playing a recorded sound and real noisy situation at home, this approach has been used in several studies to categorize the response of dogs to these sounds since it allows a more reliable analysis of the response of individual dogs without the confounding factors of home context [6, 10–12, 24–28]. Laboratory testing can be advantageous to analyse physiological and ethological measures to study the progression and nature of the sound sensitivity in dogs and to test treatment strategies.

Besides the behavioural evaluation, the present study carried out an HRV analysis which suggested that sound sensitive dogs had an exacerbated autonomic response. Changes in several different HRV parameters have been related to physical, pathological or emotional stress [29, 30]. In healthy dogs, HRV analysis is useful to estimate the emotional state of the animal. An increase in HR and LF/HF ratio are associated with emotional arousal [29, 30] with positive or negative valence. The measures of HRV have also been used to address the effect of different types of music on stress levels of sheltered dogs and to evaluate the impact of human-dog interaction [31, 32]. The analysis of HRV has been used to measure the response to sound stimulation in non-sensitive dogs. A previous study by our group showed that thunder sound increases the power of the LF band of the cardiac interval spectrum, the LF/HF ratio and the HR, and decreases the power of the HF band of the cardiac interval spectrum [6]. In the present study, among all HRV parameters analysed, only the ratio LF/HF was different between sound sensitive and non-sensitive dogs at the moment of the sound (Fig 2, panel A). Wormald and colleagues showed that dogs affected by anxiety-related behavioural problems had reduced HRV during manual restraint when compared to unaffected dogs: with a lower standard deviation of RR intervals (SDNN), high frequency (HF) spectrum and low frequency (LF) spectrum power. Unfortunately, there is no discussion about the LF/HF ratio in Wormald's paper [5]. In recent years, the LF/HF ratio has received censure as a measure of cognitive and physical aspects of stress, mainly because some authors believe that the sympathovagal balance cannot be quantified by a single number and the LF/HF “assumes a simplistic linear relationship between the activity of the nervous systems and the frequency bands" [33, 34]. Although we agree with this criticism, in the present study, the ratio LF/HF was used combined with behavioural and endocrine measures, which provides a more comprehensive view of the stress response. In other HRV parameters, sound sensitive dogs presented, independently of the sound stimulus (and in basal condition) higher HR than non-sensitive dogs and no statistical difference in SDNN and RMSSD parameters. However, there are controversies about the meaning of the RMSSD and SDNN during stress and in the basal situation, since some studies reported a decrease in RMSSD and HF for a more favourable valence of the emotional state [35, 36], while another study related a reduction in RMSSD in a negative situation and a decrease in SDNN associated with a positive condition [37]. Furthermore, although sound sensitive dogs showed a tendency to present higher SDNN/RMSSD during sound than non-sensitive, the difference was not statistically significant. Thus, the ratio LF/HF was the most consistent HRV parameter to show discrepancies between sound sensitive and non-sensitive dogs during the sound stimulus applied here.

Differently, from HRV, cortisol analyses have been used mainly as a tool to validate stress models. The analysis of cortisol has not been used as a sensible tool for comparing the magnitudes of different types of stress or analysing the effect of anti-stress strategies. While some studies have shown an apparent increase of cortisol levels in response to sound stress [13, 38], other studies have failed to show any increase [14] or this elevation was not observed in all the experimental groups[6, 16]. In the present study, although the sound increased the cortisol levels, there was no statistical difference between sound sensitive and non-sensitive dogs. The lack of difference between the groups of dogs, at least in part, was due to the substantial individual variation at basal levels, since the basal levels were used as a covariate in the statistical analysis. Furthermore, the limited number of animals also contributed to this result. We expected that the sound sensitive dogs would have a higher cortisol response. Dreschel and colleagues tested the effect of thunder sound in thunder-anxious dogs with their caregivers at home. The sound stimulus significantly increased the cortisol levels when compared to a situation of when a non-sound stimulus was applied [24]. Another important point about cortisol levels is that the results do not indicate a positive or negative valence of a reaction to the environmental stimulus, and a range of other measures are necessary to evaluate emotional distress [39].

Another aspect that should be addressed is the stressor effect of blood collection. It is well known that venous blood collection can produce changes in the cortisol levels, in behavioural and HRV parameters. In the present study, the first blood collection was performed with the animals in their respective houses. As the cortisol peak in dogs is 15 to 30 min after the stimulus, this first collection (Basal 1) was not influenced by venipuncture. The chest strip for RR interval data acquisition was placed before venipuncture. Consequently, HRV data assessed at basal 1 moment was not affected by blood collection. The second blood collection was performed with the animals in the experimental room (Basal 2). The cortisol levels at basal 2 moment had a slight increase in relation to basal 1, but this increase, in addition to not being statistically significant, cannot be solely associated with venipuncture, but also with other stimuli such as transport. The LF/HF and other HRV parameters at basal 2 moment, were not different from basal one 1 either. Thus, even recognising the blood collection as a stressor, the venipuncture did not significantly influence the parameters studied.

The sound stimulus can produce a clear behavioural response in dogs, even in non-sensitive dogs as alert and attention, panting, search sound, startle, trembling and hiding. Sound sensitive dogs have shown a more pronounced response to the parameters alert and attention, search sound, trembling, hiding and less intense response in the parameters rest and wink/sleep. Some studies have assessed several behavioural parameters in beagles or in non-sound-sensitive dogs submitted to a sound-stress model in the laboratory [6, 12–16]. Our previous study showed that the thunderstorm stimulus could induce reactions of vigilance (alert and attention), trembling, hiding and restlessness (ambulation) in laboratory dogs and domestic dogs with no history of sensitivity to sounds [6]. Few studies have addressed the effect of noise recordings in sound sensitivity dogs. Dreschel and Granger (2005) compared the impact of thunder sound with no stimulation in thunder-anxious dogs at their home environment and observed a higher occurrence of the parameters salivation, vocalisation, hiding, panting, trembling and interaction with the owner [11]. Therefore, behavioural analysis can be considered very complex in both experimental and clinical settings since there is a vast individual difference in the behavioural responses. Even in sound sensitive dogs, the behavioural response can be variable, with some dogs showing signs of arousal and little signs of fear, while others respond with noticeable signs of fear and scarce signs of arousal. Therefore, the behavioural analysis still involves the measurement of a large number of behavioural variables.

### Scoring autonomic, endocrine and behavioural parameters to build an index of sound sensitivity in dogs

Previous studies have used scores to classify the behaviour in response to a sound stimulus. In the study of Dreschel and colleagues (2005) with sound sensitive dogs, reactions such as salivation, vocalization, hiding, pacing, panting, remaining close to the owner, and trembling were graded on a scale of 1–5 based on the severity or amount observed during playing a thunderstorm recording at home (1, small amount / not severe, to 5, extensive amount, very serious). The dog's behavioural score was calculated from the sum of the scores for whining, hiding, pacing, panting, remaining close to the owner [24]. Landsberg and colleagues (2015), playing thunderstorm recordings for beagles created a global score, based on the classification of the behavioural response in: positive score, with behaviours that increase in response to sound (startle, scan (orient), bolt; aimless, repetitive or stereotypic pacing, running, or circling; digging, climbing, jumping, barking), and negative score with behaviours associated with suppression of activity and triggering of an autonomic response (freeze-against wall-at door; crouch (cower), tail between legs, ears back and pant, shake (tremble), alert/tense/vigilant, salivate, yawn, lick lip, lift foreleg, whine) [16]. Other studies have also grouped the behaviour in active and inactive anxiety signs and created a global score, where active anxiety-associated behaviours included startling; bolting; vigilance; scanning; and active responses, such as pacing, aimless activity, stereotypic circling etc.; and inactive anxiety-associated behaviours included decreased activity, such as freezing; positioning in corners, against the wall, or at door; lowered body postures, such as crouching, tail tucking, and ears back; and autonomic/conflict behaviours, such as panting, shaking, salivating, yawning, licking lips, or elimination [14, 15].

In the present study, twenty-one behaviours were grouped into four categories (arousal, fear and lack of relaxation), trying to relate each behaviour to its neurobiological/psychobiological meaning. The arousal category included active behaviours frequently associated to an aversive, novel or suddenly situation, without being specifically related to fear, like the behaviours alert and attention, panting, ambulation, search sound, startle. The fear behaviour category included classical behaviours associated to fear and were grouped as passive behaviours such as trembling, tail between legs, arched posture and freezing and as active behaviours such as running around, hiding and whining. In the relaxation category, passive behaviours were related to sleepiness and repose, such as rest and blink sleep and behaviours related to happiness in dogs as wagtail. These behaviours are typically reduced in a stress situation. Other behaviours that are frequently described in sound sensitive dogs such as yawn, bark, growl, elimination, licking lips and destruction were grouped as "others" since they are not strongly associated with any of the previous categories. The classification of the behaviours into categories has a limitation since dogs have large behavioural repertories and can learn to express specific behaviour to obtain attention from their caregivers. One example of this is the wagging tail, typically associated with happiness can also be shown during an aversive situation. Furthermore, behavioural responses to stress are interlinked and share neural pathways. Therefore, the categories cannot be related to specific neuroanatomical pathways.

To create a score of global reaction and homogenise the magnitude of the autonomic, endocrine and behavioural response, each behavioural parameter, cortisol levels and LF/HF analysis were transformed in scores (Table 2). This method is commonly used to create sustainable indicators by aggregating a set of sustainable development indicators in thematic and dimensional indices [40, 41]. Thus, this method allows the analysis of each parameter separately, in categories and with all the parameters together. In this way, each behavioural type (arousal, fear, relaxation and other), LF/HF and cortisol levels were correlated to the caregiver’s perception of the fear of the dogs at home. A moderate correlation was found between the caregiver’s perception of the dogs’ sound sensitivity and the behavioural categories arousal and fear and a high correlation between the caregiver’s perception of the dogs’ sound sensitivity and the behavioural category lack of relaxation. These results reinforce the idea that testing in a controlled environment, the sound sensitivity dogs will have more intense responses than non-sensitive dogs in the parameters autonomic (LF/HF ratio) and behavioural categories of arousal, fear, lack of relaxation. Unfortunately, despite the evidence and because of the limited number of animals, we could not run the linear regression to show that high scores in the behavioural categories arousal, fear and lack of relaxation and in the HRV parameter ratio LF/HF are predictors of sound sensitivity in dogs. After that, not only the short index for sound sensitivity (only behavioural categories arousal, fear and relaxation and LF/HF ratio were included) but also the full index for sound sensitivity (all behavioural categories, LF/HF and cortisol were included) were highly correlated to sound fear at home. These indexes can be used in future research to help diagnose sound sensitivity and can be used to evaluate treatment strategies. Moreover, the short index has an advantage as compared to the full index since it does not include the cortisol measures and therefore does not require blood collection and thus simplifies the analysis. Another important characteristic of these analyses evaluates the dog's response to a sound stimulus in the laboratory, and it is based exclusively on the dog's response, excluding the caregiver evaluation or the dog-caregiver relationship. In a clinical condition, the evaluation of the owner is fundamental for the diagnosis of behavioural disorders in pets. However, it is well known that this assessment is not based solely on the behaviour of the dog itself, but also on the owner's expectation regarding animal behaviour, which can often be unrealistic.

## Conclusion

The combined analysis of behavioural, endocrine and autonomic data allows a global and integrated view of the fear response of sound sensitive dogs. Although the diagnosis of sound sensitivity in dogs is based on behavioural responses and physiological data, it can be extremely useful in understanding the neurophysiological mechanisms of fear responses and their possible consequences for the health and well-being of the individual.

Therefore, despite the limited number of animals used, this article presents indexes of sound sensitivity that merge behavioural, endocrine and autonomic responses into a single score value. This association can be very advantageous because it represents a quantitative and less subjective analysis than the behavioural evaluation alone. Both the short and full indexes for sound sensibility can be valuable tools in future studies concerning neurophysiological mechanisms and treatment strategies of sound sensitivity in dogs.

## Supporting information

S1 Appendix General behavioural form in English. (DOCX)

S2 Appendix General behavioural form in Portuguese. (DOCX)

S3 Appendix Sound sensitive form. Owner’s Perception of dogs fear in English.
(DOCX)

S4 Appendix Sound sensitive form. Owner’s Perception of dogs fear in Portuguese.
(DOCX)

S5 Appendix Dogs information. (DOCX)

S1 Dataset Data of all animals. (XLSX)
