# Supplementary material for: Use of behavioural and physiological responses for scoring sound sensitivity in dogs
Source: PLoS One. 2018 Aug 1;13(8):e0200618. doi: 10.1371/journal.pone.0200618 (PMC6070191; doi:10.1371/journal.pone.0200618)
Supplement: S1 Appendix — (DOCX) [file pone.0200618.s001.docx]

|  | **Federal Rural University of Rio de Janeiro**  **Institute of Biological Sciences and Healthy**  **Department of Physiological Sciences** |
| --- | --- |

**Dogs general information no. _________**

| Owner: | E-mail: | |
| --- | --- | --- |
| Address: | | |
| District: | City: | Zip: |
| Home phone: | Cell phone: |  |

**Family Data:**

| Marital status: | Children: ( ) YES ( ) NO |  |
| --- | --- | --- |
| Profession: | Schooling: ( ) 1st GRADE ( ) 2nd GRADE ( ) University Graduate | |

**Animal Identification**

| Pet’s name | Dog ( ) Cat ( ) | | |
| --- | --- | --- | --- |
| Breed: | Coat: | Sex: (M) (F) | Weight: |
| Neutering? ( ) YES ( ) NO At what age Reason for castration: | | | |
| At what age did you obtain the pet? | | | |
| Where did you get your pet?   \| ( ) Found on the street \| ( ) Creator / Kennel \| ( ) Animal Shelter \| \| --- \| --- \| --- \| \| ( ) Adoption \| ( ) Born at home \| ( ) Other Which? \| \|  \|  \|  \| | | | |
| Why did you get this animal? | | | |
| Why did you choose this litter dog? | | | |
| Why did you choose this breed? | | | |
| Have you raised animals before? ( ) Yes ( ) No Which one? | | | |

**Pet’s General Routine Management:**

| Access to the house: ( ) YES ( ) NO | Access to the street: ( ) YES ( ) NO | |
| --- | --- | --- |
| Time spent indoors: % outdoors: Is this pet left alone during the day?  How long? | | |
| You allow your dog:   \| ( ) Run free with no guide on the street \| ( ) Run free in the kennel / yard / house \| ( ) Walking with leash \| \| --- \| --- \| --- \| \| ( ) Walk without a leash with your supervision \| ( ) Only in the house \|  \| | | |
| Where does your animal sleep (Check all the options, we know that the animals move at night )   \| ( ) On top of your bed \| ( ) In the kennel \| ( ) On his bed in his room \| \| --- \| --- \| --- \| \| ( ) In another room \| ( ) In the yard \| ( ) Other \| | | |
| What type of dwelling do you live in?   \| ( ) Apartment \| ( ) Town House / Condo \| ( ) Site / Farm \| \| --- \| --- \| --- \| \| ( ) House with small yard \| ( ) House with large backyard \| ( ) Other \| | | |
| Environment: | Bath: (freq., place, products, etc ) | |
| Hair brushing: |  |  |
| Diet type: Frequency: Amount: | | |
| Supplementation: ( ) YES ( ) NO | | |
| Do you have other animals? ( ) YES ( ) NO Which one (s)? | | |

**Clinical history:**

Has any behaviour changed after castration? ( ) YES ( ) NO

If the animal is not castrated, do you plan to breed it? ( ) YES ( ) NO

Has your dog crossed? ( ) YES ( ) NO

If already castrated and female, how many “heat cycle” occurred before gestation?

How old is the 1st heat? ___________

Has your dog had other owners? ( ) YES ( ) NO

How many? ( ) 1 ( ) 2 ( ) 3 ( ) 4 ( ) Unknown

Why was it donated? _______________________________

Date of last visit to veterinarian: _____________ Reason: ____________

Vaccinated? ( ) YES ( ) NO Which vaccine (s)? _____________________

Wrangler ? ( ) YES ( ) NO

Fleas ( ) YES ( ) NO Ticks? ( ) YES ( ) NO

Do you use medication (s)? ( ) YES ( ) NO Which? _______Since? ____

Have you ever had any surgery? ( ) YES ( ) NO Which one (s)? ___________________

Have you tried addressing the behavioral problem? ( ) YES ( ) No

Describe, in detail, how you prepare to leave the house when the pet will be left alone. Do you ignore your animal, you look for it and say goodbye, do you show an exaggerated affection for it?

**Indicate the behaviors that the dog presents:**

| 1 - Excretion | 4 - Run away | 7 - Shaking | 10 - Itchiness | 13 - Fighting |
| --- | --- | --- | --- | --- |
| 2 - Jump | 5 - Shyness | 8 - Biting | 11 - Digging | 14 - Howl |
| 3 - Aggression | 6 - Gnaw | 9 - Disobeying | 12 - Beating | 15 - Coprophagy |

Others? ________________________________________________________________________________

How often does it occur? ( ) Frequent (daily) ( ) Occasional Since when? _____________

Do you think that the appearance of the behavioral problem was motivated by some fact (sounds, strange people)? ( ) YES ( ) NO Which one? ____________

Has there been a change in your home since you acquired this animal? ( ) Yes ( ) No

If so, which one?

| ( ) Death of a family member | ( ) Death of a family animal | ( ) Divorce |
| --- | --- | --- |
| ( ) Marriage | ( ) Birth of baby | ( ) Moved Child |
| ( ) Animals added | ( ) Moving house | ( ) Others |
| ( ) Routine of the family changed (loss or gain of employment) |  |  |

**Current Issue:**

1. Main Complaint:
2. History:

3) Is your dog afraid of sounds of thunder and / or fireworks? How does it behave at the moment of sound? Is he more afraid of one than the other?
