# Supplementary material for: Use of behavioural and physiological responses for scoring sound sensitivity in dogs
Source: PLoS One. 2018 Aug 1;13(8):e0200618. doi: 10.1371/journal.pone.0200618 (PMC6070191; doi:10.1371/journal.pone.0200618)
Supplement: S2 Appendix — (DOCX) [file pone.0200618.s002.docx]

|  | **Universidade Federal Rural do Rio de Janeiro**  **Instituto de Ciências Biológicas e da Saúde**  **Departmento de Ciências Fisiológicas** |
| --- | --- |

**Informações gerais do animal ficha no. ________**

| Tutor: | | E-mail: |
| --- | --- | --- |
| Endereço: | | |
| Bairro: | Cidade: | Cep: |
| Tel casa: | Tel Cell: |  |

**Dados da Familia:**

| Estado civil: | Crianças: ( ) SIM ( ) NÃO |  |
| --- | --- | --- |
| Profissão: | Escolaridade: ( ) 1º. grau ( ) 1º. grau ( ) SUPERIOR | |

**Identificação do Animal**

| Nome do animal: | | Cão ( ) Gato ( ) | |
| --- | --- | --- | --- |
| Breed: | Pelagem: | Sex: ( )M ( ) F | Peso: |
| Castrado? ( ) SIM ( ) NÃO Idade da castração: Razão da castração: | | | |
| Qual idade seu pet tinha quanto você o adquiriu? | | | |
| Onde adquiriu seu pet?   \| ( ) resgatado rua \| ( ) canil/criador \| ( ) abrigo \| \| --- \| --- \| --- \| \| ( ) adoção \| ( ) nascido em casa \|  \| \| ( ) outro qual? \|  \|  \| | | | |
| Porque escolheu este animal? | | | |
| Porque escolheu esta ninhada? | | | |
| Porque escolheu esta raça? | | | |
| Já criou animais antes? ( ) SIM ( ) NÃO Qual? | | | |

**Rotina do animal:**

| Acesso a casa: ( ) SIM ( ) NÃO | | Acesso a rua( ) SIM ( ) NÃO |
| --- | --- | --- |
| Tempo dentro de casa: % Fora de casa: O animal é deixado sozinho durante o dia? Quanto tempo? | | |
| Voce permite seu cão:   \| ( ) correr livre na rua sem guia \| ( ) correr livre no canil/ quintal/casa \| ( ) andar na guia \| \| --- \| --- \| --- \| \| ( ) andar sem guia com supervisão \| ( ) so fica em casa \|  \| | | |
| Onde seu cão dorme (marque mais de uma opção caso o cão se mova a noite)   \| ( ) na sua cama \| ( ) no canil \| ( ) na cama dele \| \| --- \| --- \| --- \| \| ( ) em outro quarto \| ( ) no quintal \| ( ) Outro \| | | |
| Qual tipo de residencia ele vive?   \| ( ) Apartamento \| ( ) Casa com quintal grande \|  \| \| --- \| --- \| --- \| \| ( ) Casa com quintal pequeno \| ( ) Sítio / Fazenda \| ( ) Outro \| | | |
| Ambiente: | Banho: (freq., local, produtos, etc ) | |
| Escovação: | | |
| Tipo de Dieta: Frequência: Quantidade: | | |
| Suplementação: ( ) SIM ( ) NÃO | | |
| Voce tem outros animais? ( ) SIM ( ) NÃO Quais (s)? | | |

**História clínica:**

Algum comportamento mudou após a castração? ( ) SIM ( ) NÃO

Se o animal não é castrado, você planeja que ele procrie? ( ) Sim ( ) Não

Seu cão já cruzou? ( )Sim ( ) Não

O seu cão teve outros donos? ( ) Sim ( )Não

Quantos? ( )1 ( )2 ( )3 ( )4 ( )Desconhece

Porque ele foi doado? _______________________________

Data da última visita ao veterinário: _____________Motivo:____________

2) Vacinado? ( ) SIM ( ) Não Qual(is) vacinas? _____________________

Vermifugado? ( ) SIM ( ) Não

Pulgas? ( ) SIM ( ) Não Carrapatos? ( ) SIM ( ) NÃO

Faz uso de medicamento(s)? ( )SIM ( ) NÃO Qual? _______Desde? ____

Já fez alguma cirurgia? ( ) SIM ( ) NÃO Qual(is)?___________________

Possui outros animais? ( ) SIM ( ) NÃO Qual(is)?___________________

Já tentou tratar o problema comportamental? ( ) SIM ( ) Não

Descreva, com detalhes, como você prepara para sair de casa quando o animal vai ficar sozinho. Você ignora seu animal, você busca por ele e se despede, você demonstra um afeto exagerado por ele?

**Comportamentos observados no cão:**

| 1 – Excreção | 4 – Fugir | 7 – Agitação | 10 – Pica | 13 – Brigar |
| --- | --- | --- | --- | --- |
| 2 – Pular | 5 – Timidez | 8 – Morder | 11 – Cavar | 14 – Uivar |
| 3 – Agressão | 6 – Roer | 9 – Desobedecer | 12 – Latir | 15– Coprofagia |

Outros? ______________

Com que frequência ele ocorre? ( ) Frequente (diário) ( ) Ocasional

Desde quando? ______

Você acha que o aparecimento do problema comportamental foi motivado por algum fato (sons, pessoas estranhas)? ( ) SIM ( ) Não Qual? ____________

Houve mudança na sua casa desde que adquiriu este animal? ( ) SIM ( ) NÃO

Se sim qual?

| ( ) Morte de pessoa da família | ( ) Morte de animal da família | ( ) Divórcio |
| --- | --- | --- |
| ( ) Casamento | ( ) Nascimento de bebê | ( ) Mudança de uma criança |
| ( ) Animais adicionados | ( ) Mudança de casa | ( ) Outros |
| ( ) Rotina da família alterada (perda ou ganho de emprego) |  |  |

**Questão atual:**

1. Queixa Principal:
2. Histórico:
3. Seu cão tem medo de sons de trovão e/ou fogos de artifício? Como ele se comporta no momento do som? Ele tem mais medo de um do que do outro?
