# Supplementary material for: Use of behavioural and physiological responses for scoring sound sensitivity in dogs
Source: PLoS One. 2018 Aug 1;13(8):e0200618. doi: 10.1371/journal.pone.0200618 (PMC6070191; doi:10.1371/journal.pone.0200618)
Supplement: S3 Appendix — Owner’s Perception of dogs fear in English. (DOCX) [file pone.0200618.s003.docx]

| **** | **Federal Rural University of Rio de Janeiro**  **Institute of Biological Sciences and Healthy**  **Department of Physiological Sciences** |
| --- | --- |

**Sound sensitive form**

**Patient name:_______________________________________**

**Owner name: _______________________________________________________________**

Please check all behaviors that your dog exhibits during the aversive sound:

| **Destructiveness**   \| 1  Small items (pens, paper, etc.) \| 2 \| 3 \| 4 \| 5  Extensive damage (e.g. holes in wall, etc.) \| \| --- \| --- \| --- \| --- \| --- \| |
| --- | --- | --- | --- | --- | --- |
| **Elimination (circle one: urination defecation or both)**   \| 1  Small amount \| 2 \| 3 \| 4 \| 5  Extensive amount \| \| --- \| --- \| --- \| --- \| --- \| |
| **Excess salivation**   \| 1  Damp mouth \| 2 \| 3 \| 4 \| 5  Wet mouth and forepaws \| \| --- \| --- \| --- \| --- \| --- \| |
| **Excess vocalizations (during storms of >1 hour)**   \| 1  Less than 2 minutes \| 2  5-15 min. \| 3  15-30 min. \| 4  30 min. – 1 hour \| 5  More than 1 hour \| \| --- \| --- \| --- \| --- \| --- \| |
| **Hiding**   \| 1  Small amount \| 2 \| 3 \| 4 \| 5  Extensive amount \| \| --- \| --- \| --- \| --- \| --- \| |
| **Pacing**   \| 1  Small amount \| 2 \| 3 \| 4 \| 5  Extensive amount \| \| --- \| --- \| --- \| --- \| --- \| |
| **Panting**   \| 1  Small amount \| 2 \| 3 \| 4 \| 5  Extensive amount \| \| --- \| --- \| --- \| --- \| --- \| |
| **Remains near owner**   \| 1  Small amount \| 2 \| 3 \| 4 \| 5  Extensive amount \| \| --- \| --- \| --- \| --- \| --- \| |
| **Self-trauma**   \| 1  Small amount (licking feet, etc.) \| 2 \| 3 \| 4 \| 5  Extensive trauma (lacerations, broken teeth etc \| \| --- \| --- \| --- \| --- \| --- \| |
| **Trembling**   \| 1  Small amount \| 2 \| 3 \| 4 \| 5  Extensive amount \| \| --- \| --- \| --- \| --- \| --- \| |
| **Other:**   \| 1  Small amount \| 2 \| 3 \| 4 \| 5  Extensive amount \| \| --- \| --- \| --- \| --- \| --- \| |
| **Total** |

Crowell-Davis et al., 2003
