# Supplementary material for: Use of behavioural and physiological responses for scoring sound sensitivity in dogs
Source: PLoS One. 2018 Aug 1;13(8):e0200618. doi: 10.1371/journal.pone.0200618 (PMC6070191; doi:10.1371/journal.pone.0200618)
Supplement: S4 Appendix — Owner’s Perception of dogs fear in Portuguese. (DOCX) [file pone.0200618.s004.docx]

| **** | **Universidade Federal Rural do Rio de Janeiro**  **Instituto de Ciências Biológicas e da Saúde**  **Departmento de Ciências Fisiológicas** |
| --- | --- |

**Questionário de Sensibilidade a Sons**

**Nome do animal:_______________________________________**

**Nome do tutor: _______________________________________________________________**

Marque todos os comportamentos que seu cão apresenta durante o som:

| **Atitude destrutiva**   \| 1  Pequenos itens (caneta, papel, etc.) \| 2 \| 3 \| 4 \| 5  Grandes destruições (e.g. furos na parede, etc.) \| \| --- \| --- \| --- \| --- \| --- \| |
| --- | --- | --- | --- | --- | --- |
| **Eliminação (marque um: urina, fezes ou ambos)**   \| 1  Poucas vezes \| 2 \| 3 \| 4 \| 5  Muitas vezes \| \| --- \| --- \| --- \| --- \| --- \| |
| **Salivação excessiva**   \| 1  Boca molhada \| 2 \| 3 \| 4 \| 5  Boca e patas molhadas \| \| --- \| --- \| --- \| --- \| --- \| |
| **Vocalização excessiva (durante sons prolongados)**   \| 1  Menos de 2 m \| 2  5-15 min. \| 3  15-30 min. \| 4  30 min. – 1 hora \| 5  Mais de 1 hora \| \| --- \| --- \| --- \| --- \| --- \| |
| **Se esconder**   \| 1  Poucas vezes \| 2 \| 3 \| 4 \| 5  Muitas vezes \| \| --- \| --- \| --- \| --- \| --- \| |
| **Andar**   \| 1  Poucas vezes \| 2 \| 3 \| 4 \| 5  Muitas vezes \| \| --- \| --- \| --- \| --- \| --- \| |
| **Arfar**   \| 1  Poucas vezes \| 2 \| 3 \| 4 \| 5  Muitas vezes \| \| --- \| --- \| --- \| --- \| --- \| |
| **Permanecer perto do dono**   \| 1  Poucas vezes \| 2 \| 3 \| 4 \| 5  Muitas vezes \| \| --- \| --- \| --- \| --- \| --- \| |
| **Auto-mutilação**   \| 1  Poucas vezes (lamber pata, etc.) \| 2 \| 3 \| 4 \| 5  Trauma extensor (lacerações dente quebrado etc \| \| --- \| --- \| --- \| --- \| --- \| |
| **Tremor**   \| 1  Poucas vezes \| 2 \| 3 \| 4 \| 5  Muitas vezes \| \| --- \| --- \| --- \| --- \| --- \| |
| **Outro:**   \| 1  Poucas vezes \| 2 \| 3 \| 4 \| 5  Muitas vezes \| \| --- \| --- \| --- \| --- \| --- \| |
| **Total** |

Crowell-Davis et al., 2003
