# Supplementary material for: Use of behavioural and physiological responses for scoring sound sensitivity in dogs
Source: PLoS One. 2018 Aug 1;13(8):e0200618. doi: 10.1371/journal.pone.0200618 (PMC6070191; doi:10.1371/journal.pone.0200618)
Supplement: S5 Appendix — (DOCX) [file pone.0200618.s005.docx]

Supplementary data:

Table 1. Dogs general characteristic and owner’s perception of sound sensitivity.

| Pet’s Name | Weight (kg) | Age  (years) | Sex | Spayed | Breed | Sound sensitivity  score |
| --- | --- | --- | --- | --- | --- | --- |
| Non-sensitive dogs | | | | | | |
| Amim | 15 | 4 | M | NO | Mixed Breed | 0 |
| Darwin | 28 | 3 | M | NO | Dalmatian | 0 |
| Nina | 30 | 5 | F | YES | Mixed Breed | 0 |
| Roseta | 16 | 2 | F | NO | Mixed Breed | 1 |
| Yelow | 12 | 3 | F | YES | Mixed Breed | 2 |
| Axel | 23 | 3 | M | NO | Mixed Breed | 2 |
| Sound-sensitive dogs | | | | | | |
| Belinha | 15 | 3 | F | YES | Mixed Breed | 23 |
| Katira | 27 | 2 | F | NO | Australia Sheppard | 26 |
| Laila | 12 | 2 | F | YES | Mixed Breed | 28 |
| Liz | 10 | 5 | F | NO | Lhasa Apso | 26 |
| Pitty | 15 | 3 | F | YES | Mixed Breed | 19 |
| Preta | 12 | 3 | F | NO | Mixed Breed | 18 |
| Susy | 10 | 5 | F | NO | Mixed Breed | 15 |
| Toquinho | 13 | 5 | M | YES | Mixed Breed | 18 |
| Antônia | 14 | 2 | F | YES | Mixed Breed | 22 |
| Baloo | 27 | 6 | M | YES | Border Collie | 19 |
| Half | 19 | 4 | M | NO | Mixed Breed | 22 |
| Layla | 32 | 3 | F | NO | Labrador | 24 |
